# Supplementary material for: Comparative analysis of rhizosphere microbiomes of cultivated and wild rice under contrasting field water regimes
Source: Microbiol Spectr. 2025 Sep 25;13(11):e00263-25. doi: 10.1128/spectrum.00263-25 (PMC12584754; doi:10.1128/spectrum.00263-25)
Supplement: Supplemental figures — Figures S1 to S8. [file spectrum.00263-25-s0001.docx]

Exploring the role of rhizosphere microbes in wild and cultivated rice in enhancing water-limiting resistance

Yuhong Luo ^1^, Xiaolong Xu^1^, Renfei Qiao^1^, Rupeng Zhao^1^, Zu-Wen Zhou^1^, Dong-Ao Li^1^, Yuhao Wen^1^, Jia-Ming Song ^2*^, Ling-Ling Chen^1,3*^

^1^ State Key Laboratory for Conservation and Utilization of Subtropical Agro-bioresources, College of Life Science and Technology, Guangxi University, Nanning 530004, China

^2^ College of Agronomy and Biotechnology, Southwest University, Chongqing 400715, China

^3^ Yazhouwan National Laboratory, Sanya, 572025, China

*Correspondence

Jia-Ming Song, E-mail address: [jmsong@swu.edu.cn](mailto:jmsong@swu.edu.cn); Tel: +86 15827364552;

Ling-Ling Chen, E-mail address: [llchen@gxu.edu.cn](mailto:llchen@gxu.edu.cn); Tel: +86 18971629380

**Supplementary figures**


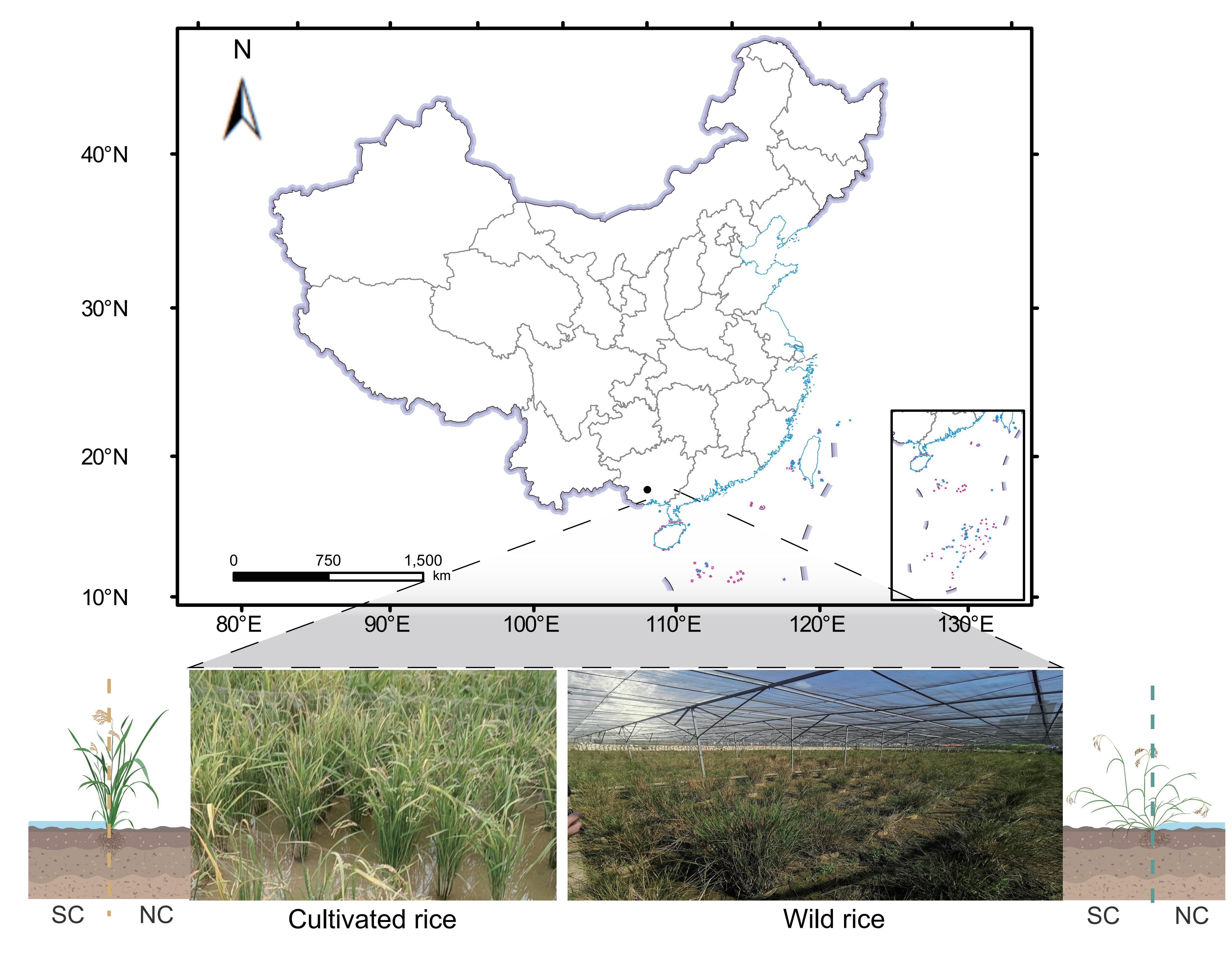


**Fig. S1 This map illustrates the sampling locations for the study, which includes the wild rice conservation field at Guangxi University and the adjacent cultivated rice planting area. The diagram also depicts wild rice and cultivated rice under non-irrigated and submergence conditions.**


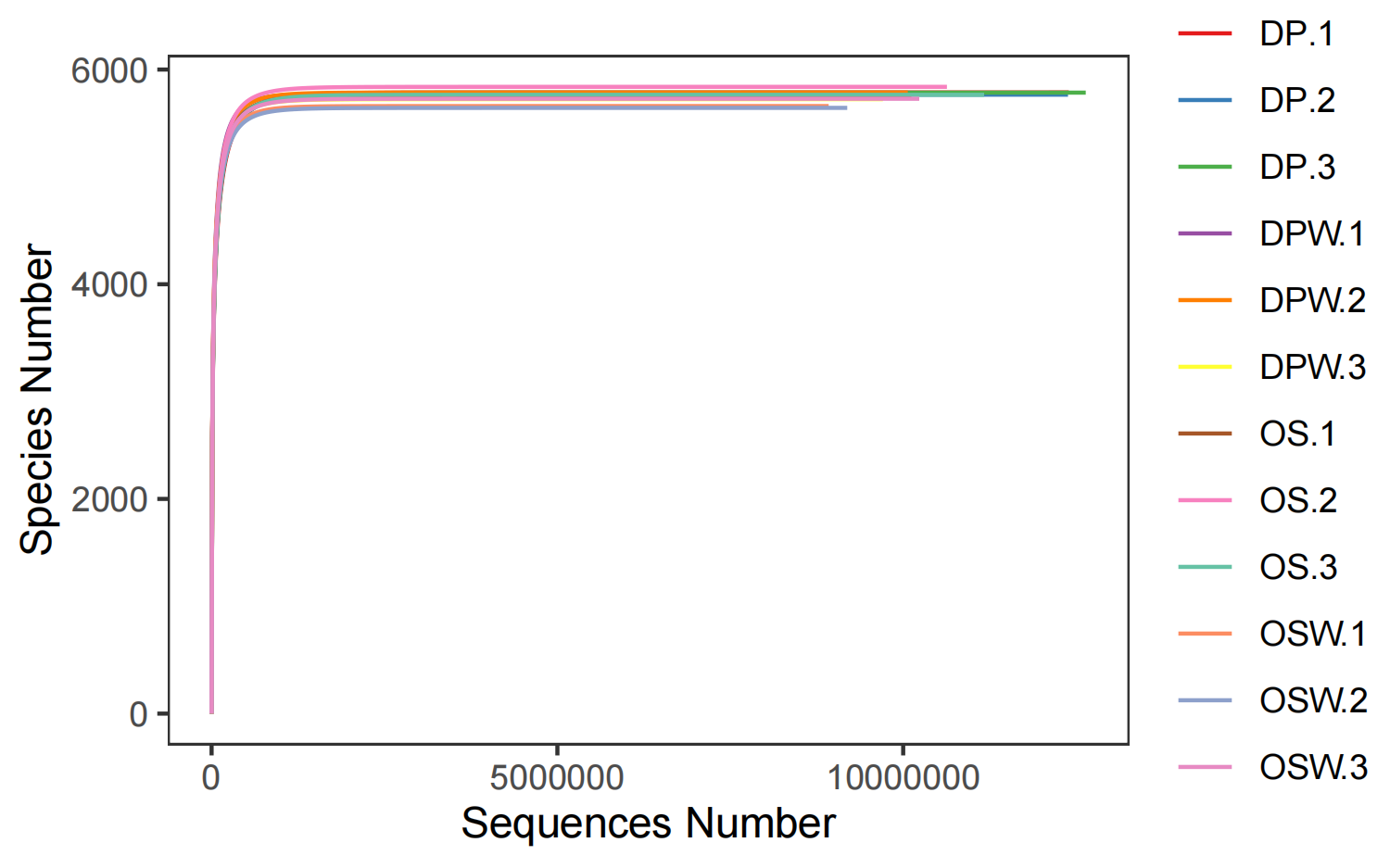


**Fig. S2.** **Rarefaction curve is based on the number of microbial species****. Each color represents one sample.**


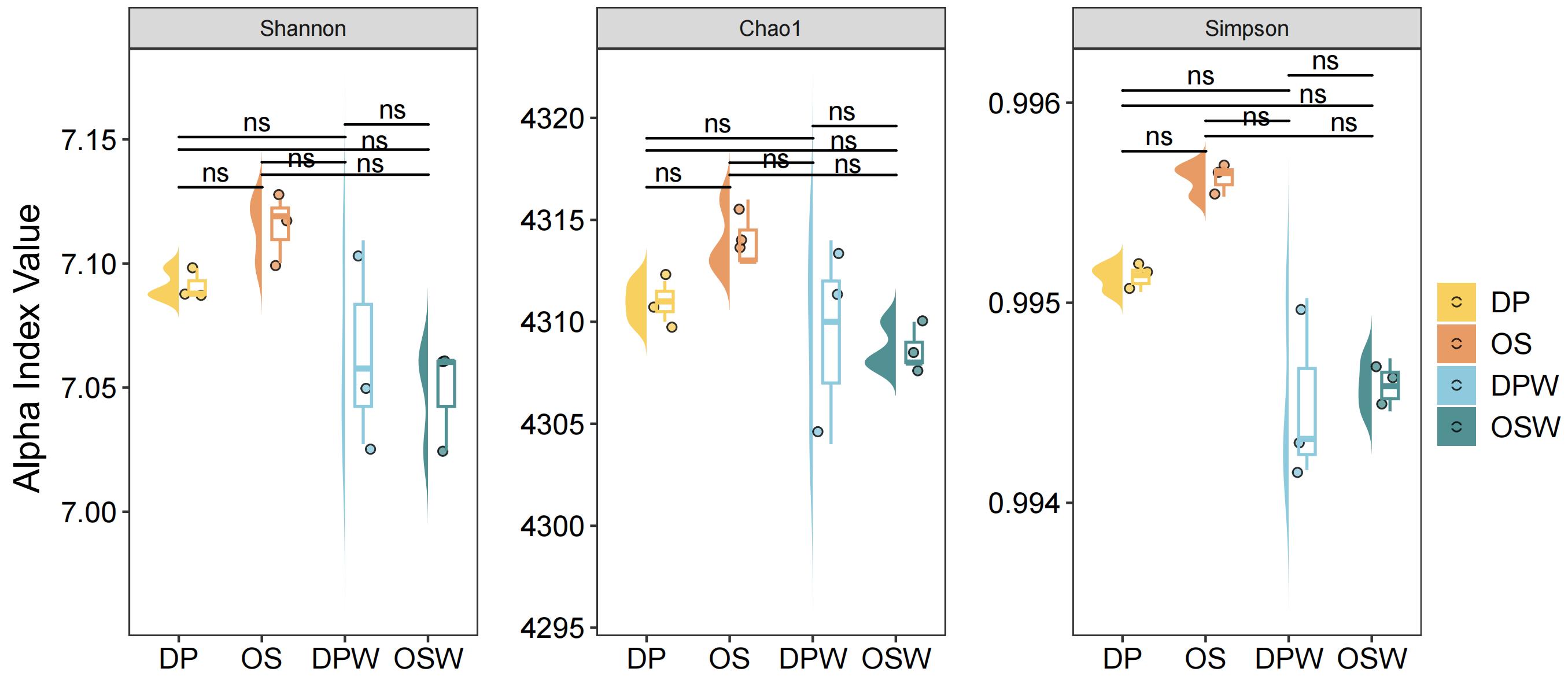


**Fig. S3** **Microbial α-diversity (****Shannon, Chao1 and Simpson) of rhizosphere soil under different conditions.** “ns” represents no significant difference between groups.


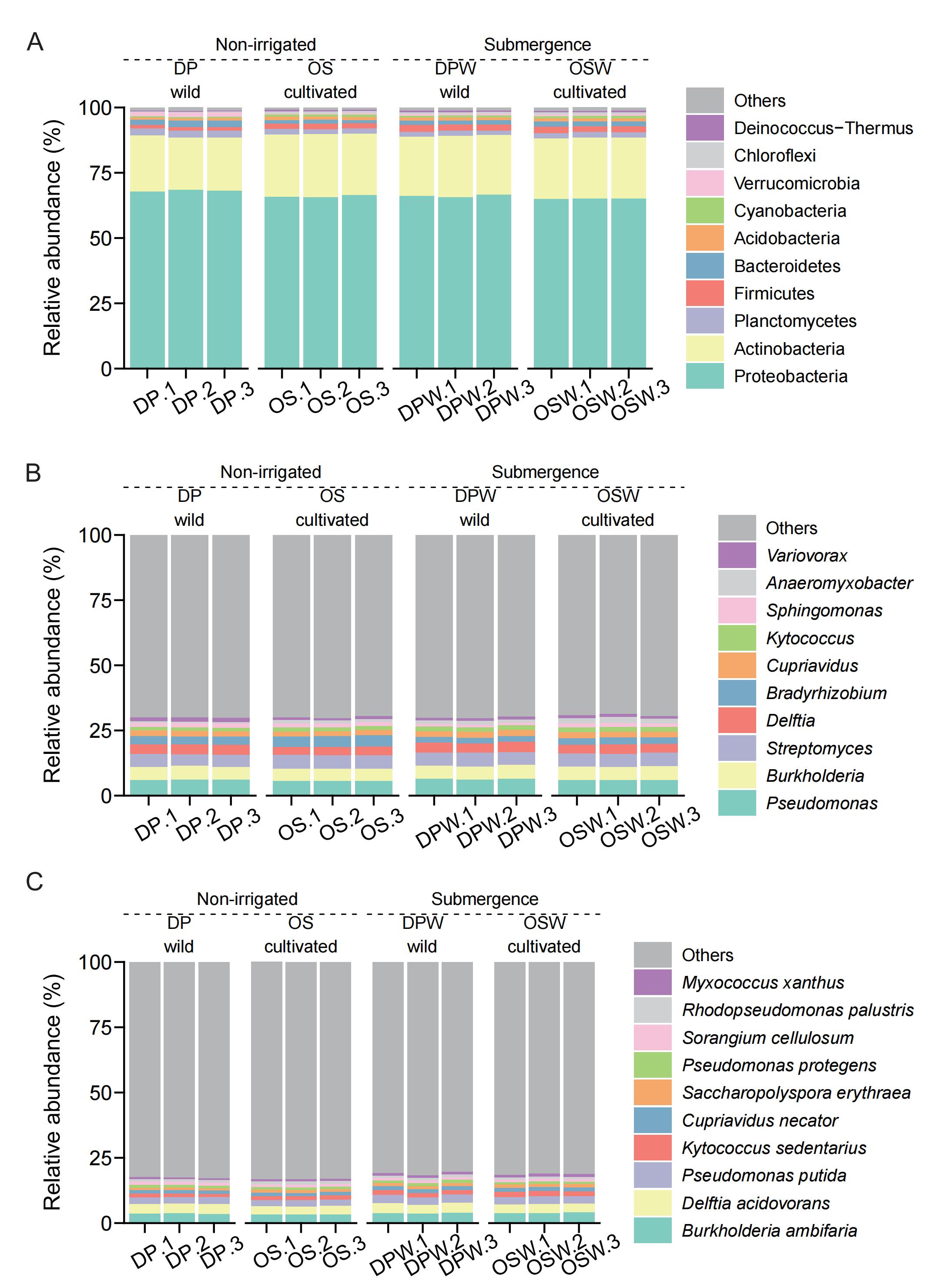


**Fig. S4**. **Relative abundance of bacterial communities in rice rhizosphere soils under different conditions at the phylum, genus, and species levels.**


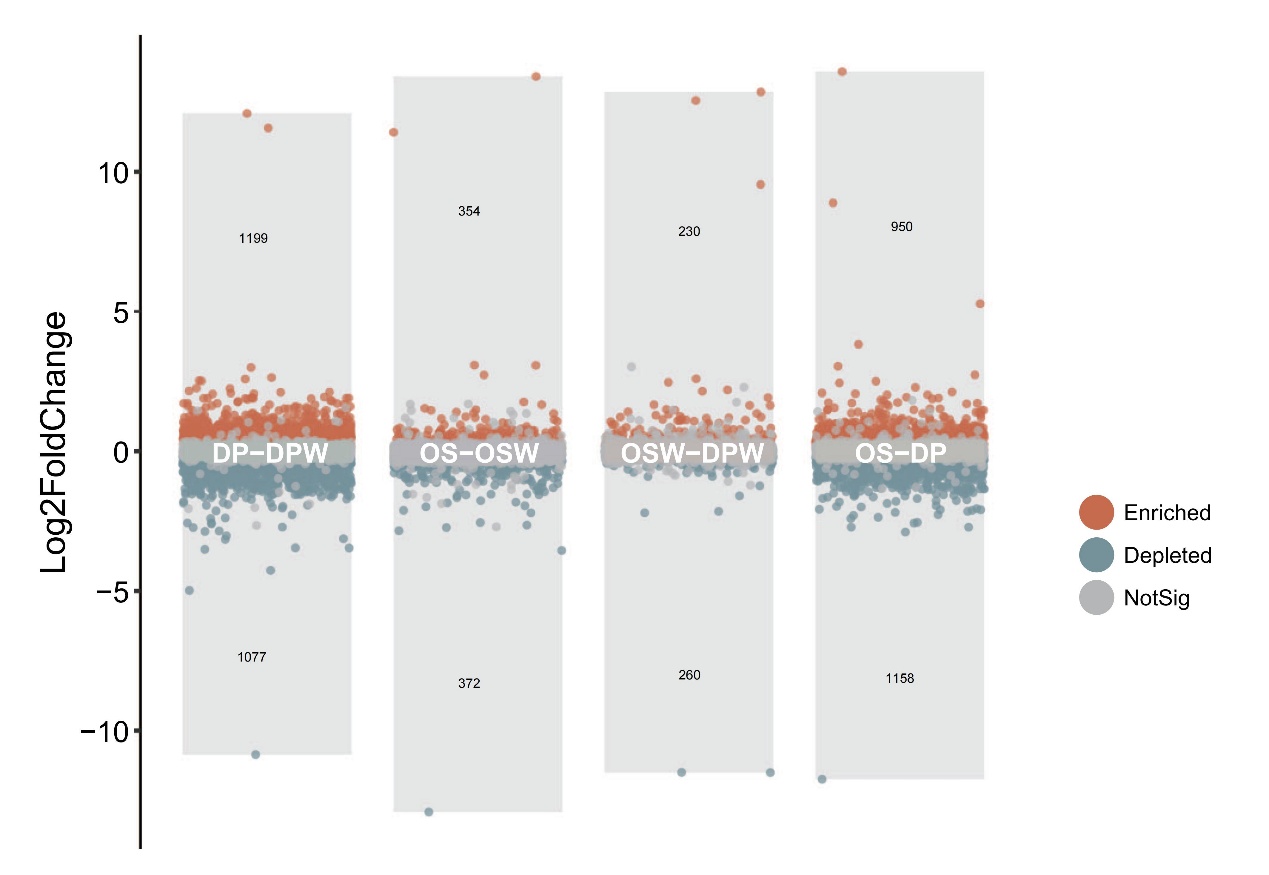


**Fig. S5** **Volcano plot showing differential microbial abundance at the species level in the rhizosphere of wild and cultivated rice under submergence and non-irrigated condition.** Blue represents depleted microorganisms, red represents enriched microorganisms, and gray represents microorganisms with no significant difference.


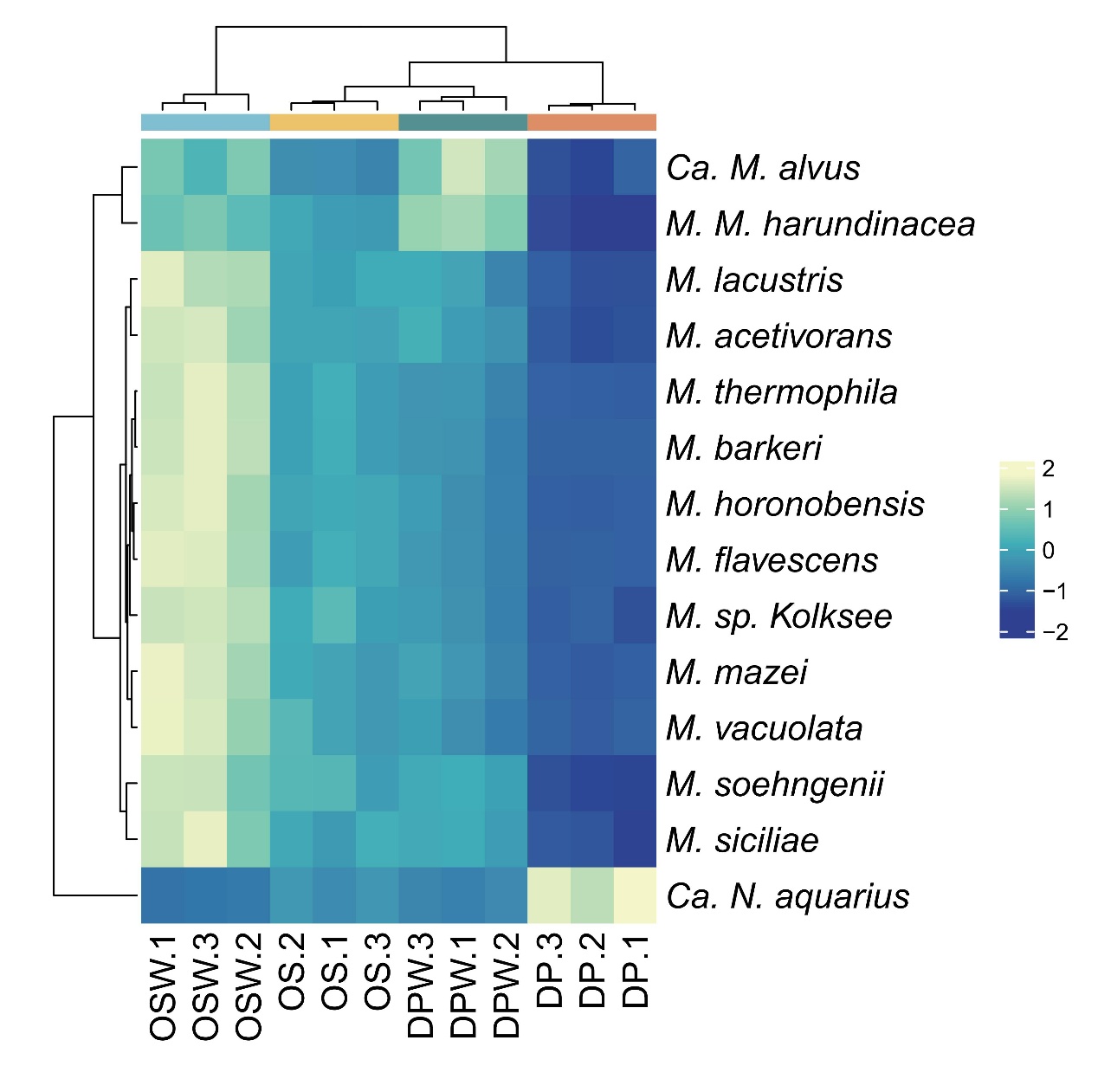


**Fig. S6** **Heatmap showing the relative abundance of differential archaea in the rhizosphere of submergence and non-irrigated conditions under both wild and cultivated rice.** The relative abundance values were scaled and displayed in varying colors, with yellow indicating high expression and blue representing low expression.


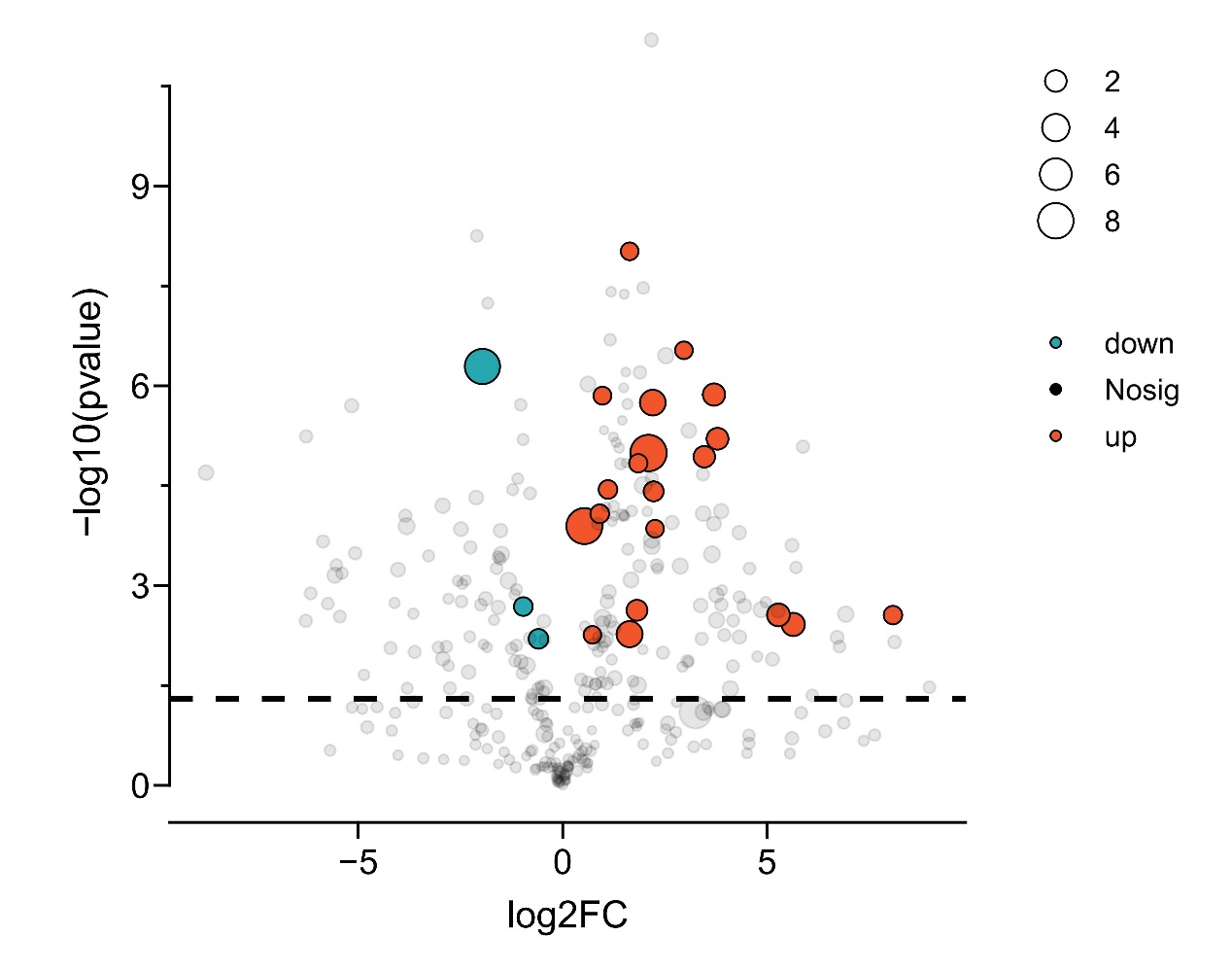


**Fig. S7** **Volcano plot depicting differential metabolite levels in the rhizosphere of wild and cultivated rice under non-irrigated conditions.** Blue represents metabolites upregulated in wild rice, red indicates metabolites upregulated in cultivated rice, and gray denotes metabolites with no significant difference. The size of the circles corresponds to the magnitude of the Variable Importance in Projection (VIP) score.


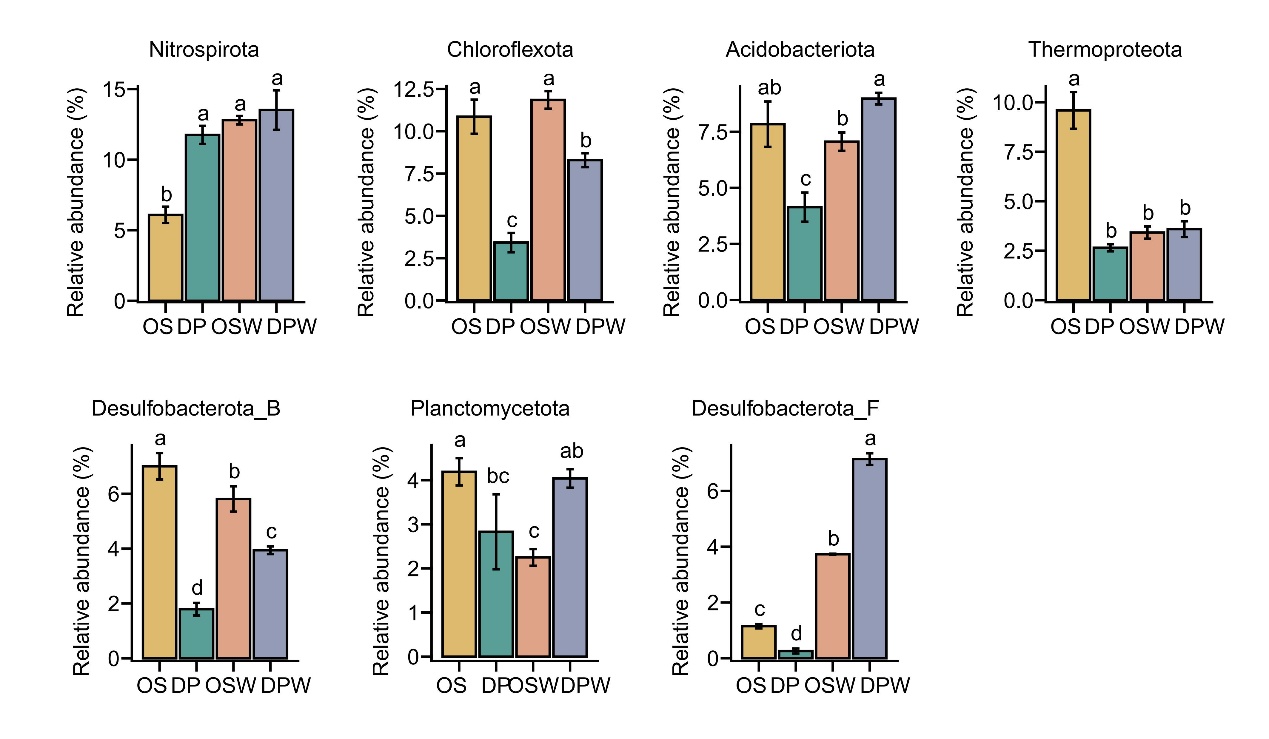


**Fig. S8** **Relative abundance of MAGs from seven bacterial phyla in rice rhizosphere soil under different conditions.** Letters represent significantly different post hoc pairwise comparisons via Tukey’s test (*P* < 0.05, n = 3).
